# Manual of Hypodermic Medication

**Published:** 1889-03

**Authors:** 


*Manual of Hypodermic Medication.* By Drs. Bourneville and Bricon. Translated from the Second Edition, with Additions by Andrew S. Currie, M.D. London: H. K. Lewis. 1887.

Dr. Currie has done good service in giving us a translation of this book, as it is most desirable that English readers should be made familiar with foreign works, that have been well received in their own country.

There is first a most meagre history of the hypodermic method and a short account of the various syringes, &c., that have been used in the procedure. The main part of the book consists of a list of drugs, in alphabetical order, giving the various preparations and doses that have been used, and the conditions in which they have been found serviceable. Some useful tables (including the growingly popular, but almost pernicious, "Index of Diseases") and a good index are appended.

The book is not a mere translation of the work of Drs. Bourneville and Bricon, as Dr. Currie frequently interposes comments of his own, based upon personal experience or culled from his research. Both authors and translator seem to have ransacked literature to find and chronicle every hypodermic "fad" of enthusiastic therapeutists. Much of this might have been omitted with advantage to the reader, and in the interests of the book itself. The excellent practice of subcutaneous medication is likely to suffer when we are gravely told by the authors of the use of "sulphate of magnesia (1½ grain in 16 minims of distilled water) by the hypodermic method, and the purgative effect was produced," or when the translator leads us to infer that, in a case of cancer of the uterus, the hypodermic use of a sedative gave relief for ten or fourteen days at a time.

The book, however, is one that all those should have who wish to know of what this important branch of therapeutics is capable.

